# Allergic asthma: An indication for allergen immunotherapy 

**DOI:** 10.5414/ALX02332E

**Published:** 2023-03-01

**Authors:** Tobias Ankermann, Randolf Brehler

**Affiliations:** 1 Childrens Hospital, Städtisches Krankenhaus Kiel gGmbH, Kiel, and; 2 Department of Dermatology, University Hospital Münster, Germany

**Keywords:** asthma, allergic asthma, allergen-specific immunotherapy (AIT), environmental allergens, meta-analyses

## Abstract

Abstract. Allergen immunotherapy (AIT) as a validated, disease-modifying treatment is nowadays a widely recommended therapy option for allergic rhinitis and allergic asthma. The registration of allergen extracts used for AIT is based on allergen standardization, dose finding trials, and phase 3 trials proving their efficacy in high-quality, statistically significant randomized clinical trials. Real-world evidence (RWE) studies confirm the clinical relevance of these findings. The most data are available for the treatment of patients suffering from allergic rhinitis. Due to the similar inflammatory mechanisms, allergic rhinitis is often associated with allergic asthma. AIT, which induces tolerance against individual allergens, is the approach to treat the underlying mechanisms of these two interrelated respiratory diseases. Some trials have been published focusing primarily on the effect of AIT on parameters of allergic asthma. Here we give a summary of the evidence for the efficacy of AIT in the indication of allergic asthma.

## Background 

Asthma, which is a heterogeneous disease characterized by chronic airway inflammation, is a global health problem effecting more than 330,000,000 people worldwide. The prevalence is increasing in many countries, and 1 – 18% of the whole population is affected in different countries [1]. Aeroallergens are responsible for IgE-mediated allergies triggering type-2 inflammation which is of fundamental importance for allergic asthma and allergic rhinitis. The prevalence of allergic sensitization against those allergens varies between 30 and 79% in children and 30 and 60% in adults [2]. 

For a long time, the importance of allergen-specific immunotherapy (AIT) to treat allergic asthma was controversially discussed; nowadays, it is recommended to take the allergy-related medical history of all asthma patients and to perform allergy tests routinely to prove allergen avoidance measures and, if indicated, as a treatment option. Patients with allergic asthma mostly suffer simultaneously from comorbidities, e.g., allergic rhinitis, food allergies, and also from atopic dermatitis. For patients suffering from allergic rhinitis, AIT is the sole evidence-based treatment option inducing tolerance against individual allergens. In these patients, AIT has sustained clinical effects, modifies underlying allergic disease mechanisms, and prevents the extension of sensitization and the development of secondary diseases. All other registered drugs used for the treatment of inhalant allergies act only in an antisymptomatic way. 

The efficacy of AIT has been proven mostly in clinical trials on patients with allergic rhinitis, and in some studies the effect on comorbid allergic asthma has been demonstrated. Subcutaneous (SCIT) and sublingual immunotherapy (SLIT) affects the inhaled corticosteroids (ICS) dose needed for asthma control; therefore, AIT is an ICS-sparing treatment. In a prove-of-concept trial, SLIT reduced the incidence of severe asthma attacks after cessation of ICS in a statistically significant way compared with placebo. 

The evidence for efficacy of AIT in patients suffering from allergic asthma is nowadays based on randomized clinical trials (RCTs) proving efficacy of several allergen extracts, and real-world evidence (RWE) studies confirm effectiveness. 

In Germany, AIT is currently recommended for allergic asthma, if it is well documented that allergens elicit asthma symptoms and if it is not uncontrolled [3]. 

## Allergens in allergic asthma 

Most patients with allergic asthma suffer from comorbid allergic rhinitis. The most important allergens triggering allergic rhinitis derive from house dust mites (HDM), pollens, pets, and molds. The inhalation of allergens induces a type-2 inflammation in sensitized individuals, leading to the symptoms of allergic rhinitis and asthma with bronchial hyperactivity, inducing inflammation and subsequently tissue remodeling due to persisting allergen exposure. 

HDM allergens are the most important perennial allergens worldwide; in Europe, ubiquitous grass pollen along with birch pollen in the northern, and olive pollen in the southern part are the most important seasonal allergens. 

## Efficacy of AIT 

Clinical trials focusing on the efficacy of SCIT and SLIT for the treatment of allergic asthma have been summarized in Cochrane analyses. 

88 SCIT trials were included in the Cochrane analyses published in 2010 [4]. Most of the studies used HDM extracts, followed by different types of pollen, animal dander, molds, latex, and some trials used multiple allergens. Overall, due to AIT, a significant reduction of asthma symptoms and asthma medication and an improvement in the bronchial hyperreactivity was observed. Treatment of 3 patients with AIT avoided a deterioration of asthma symptoms in 1. Also, a reduction of unspecific bronchial hyperreactivity was observed. AIT has been considered as a safe therapy, nevertheless in the AIT treatment of 9 patients 1 would be expected to develop a systemic reaction. 

66 SLIT studies including overall 7,944 participants and focusing on the efficacy of SLIT with various allergens (mostly HDM, in some trials grass or birch pollen, cat dander, *Alternaria*, olive, artemisia, or *Parietaria* pollen extracts) to treat mostly mild intermittent asthma were identified [5]. The study design of the included trials was highly heterogeneous; the trials recruited patients suffering from asthma, rhinitis, or both, at least 80% of the trial participants had a diagnosis of asthma. Endpoints were not uniformly defined – different symptom and medication scores were evaluated, required ICS doses, response to provocation tests, the frequency of asthma exacerbation, and in some studies quality-of-life scores. The allergen concentrations in the extracts were highly variable and not comparable. The authors concluded that SLIT is a safe treatment option for patients with well-controlled mild to moderate asthma and rhinitis, but the evidence for important outcomes such as exacerbations and quality of life was too limited to draw conclusions about the overall efficacy of SLIT in patients suffering from asthma. 

In 2017, a systematic review and meta-analysis was published, in which 98 studies were evaluated [6]. Overall, it was reported that AIT in allergic asthma can achieve a substantial reduction in short-term symptom and medication scores. Quality of life and allergen-specific airway hyperreactivity was improved only due to SCIT. 

### Reduction of symptoms and antisymptomatic drugs in clinical trials 

In a Chinese double-blind, placebo-controlled (DBPC) study, 132 asthma patients aged 6 – 45 years were treated with a *Dermatophagoides pteronyssinus* extract over 52 weeks. All patients suffered from mild to moderate asthma, all were sensitized to HDM, and 50% were polysensitized. At the end of the study, a significant reduction of medications and symptoms associated with a subjective improvement in asthma control was observed [7]. 

In a study from Germany, 65 children at the age of 6 – 17 years treated with ICS for mild to moderate HDM allergic asthma were randomized to be treated with antisymptomatic medication alone or in combination with SCIT using a HDM allergoid [8]. After 2 years, the ICS dose needed to control asthma symptoms could be reduced in the SCIT group from 330.3 to 151.5 µg and in the control group from 290.6 to 206.3 µg. The difference was statistically significant; compared with the control group, significant improvement was observed in the morning peak expiratory flow. 

The efficacy of HDM SLIT tablets on asthma symptoms and asthma medications was evaluated in a large DBPC trial on 604 subjects with controlled or partially controlled mild to moderate asthma [9]. After 1 year of treatment, there was a statistically significant reduction of the ICS dose necessary to control asthma in patients treated with HDM SLIT compared with the placebo group. 

Another HDM tablet was investigated for the treatment of allergic rhinitis in a DBPC trial on 1,670 12- to 65-year-old patients [10]. 37.6% of the patients suffered from coincident asthma. The effect of SLIT on asthma symptoms was not evaluated in this trial but is indicated by the effect on life quality assessments demonstrating no difference between patients with and without asthma. 

The EAACI guideline on AIT with HDM extracts in patients suffering from allergic asthma summarizes the literature and recommends HDM SLIT with the investigated tablet as add-on to the regular asthma therapy for adults with controlled and partially controlled HDM-driven allergic asthma. HDM SCIT was recommended for adults and children, and SLIT drops were recommended for children with controlled HDM-driven allergic asthma [11]. 

### Reduction of asthma exacerbation in clinical trials 

In a DBPC phase 3 trial on 834 HDM-allergic patients suffering from mild to moderate asthma not well controlled by ICS, the efficacy of the HDM tablet has been evaluated in regard to prevent asthma exacerbation after reduction or cessation of ICS [12]. In this proof-of-concept study, patients were treated over 1 year, then the ICS dose was halved and then completely stopped. The primary endpoint of this study was the time to the first moderate or severe asthma exacerbation during the ICS reduction. The result was statistically significant and clearly indicated that SLIT with that HDM tablet was able to reduce asthma exacerbations in patients suffering from not-well-controlled allergic asthma. Comparable trials using SCIT have not been published yet. 

### Real-world evidence for the efficacy of AIT to treat allergic asthma 

RWE studies add important information to the body of evidence given by randomized, controlled trials. Effects of AIT are evaluated in big populations and covering longer periods, they give important information about adherence, and include not only those highly selected patients treated in RCTs. Nevertheless, RWE studies use a retrospective design, and efficacy of the intervention cannot be estimated due to missing placebo groups, and clinical information must be collected by proxy [13]. Quality criteria for these trials have been created recently [14]. 

A seminal study published in 2015 used data of individuals with allergic rhinitis from a German health insurance (AOK Saxony): 2,431 patients were treated with AIT and 116,323 only with antisymptomatic drugs. Included were patients getting SCIT or SLIT with various allergens. During the observation period, overall 1,646 patients had incident asthma – 33 patients in the AIT and 1,617 in the control group. It was summarized that AIT reduces the risk for the development of asthma by 40% in patients suffering from allergic rhinitis in a real-life setting [15]. Later, the same group demonstrated that patients with allergic asthma who receive AIT (with various allergens) are less likely to experience a progression of asthma severity [16]. 

Data from a French database could demonstrate that patients receiving grass pollen SLIT (n = 1,099) had a significantly lower relative risk for new asthma and a slower progression of asthma using asthma medication as a proxy compared with 27,475 control patients [17]. 

An analysis of data from a large German prescription database (LRx) revealed that SLIT with grass pollen (n = 2,851) was associated less frequently with asthma onset and slower asthma progression with a posttreatment effect of 2 years [18]. Using sets from the same data base (LRx) for birch pollen AIT (six different AIT produccts were used; native SLIT, native SCIT, four allergoid SCITS) demonstraited that up to 6 years after AIT cessation, asthma medication and the new onset of asthma medication use was reduced due to AIT [19]. 

For patients with mite-induced allergic rhinitis who received AIT, a significant reduction of asthma medication was demonstrated recently [20]. In this cohort analyses of a German prescription database, 2,350 patients receiving HDM SCIT were compared with 64,740 control patients. During the follow-up of up to 6 years, SCIT-treated patients required significantly fewer prescriptions for allergic rhinitis and asthma medication. 

A RWE study designed to asses adherence to AIT revealed that SCIT with grass and tree pollen reduced asthma medication, whereas this was demonstrated for SLIT only with grass but not with birch pollen [21]. 

The so-far newest RWE focusing on the efficacy of AIT is based on data from a German health insurance (Betriebskrankenkasse (BKK)) [22]. 46,024 patients treated with AIT for allergic rhinitis were matched with the same number of control subjects; each 14,114 patients suffered additionally from asthma. Patients entered the trial in 2007 and were followed up over the subsequent 9 years. AIT treatment was associated with a greater reduction in prescriptions of medications for control or relieve of allergic rhinitis and asthma; the AIT cohort had a significantly greater likelihood of stepping down asthma treatment and a significantly greater reduction in severe asthma exacerbations and a reduction in pneumonias treated with antibiotics and leading to hospitalization. Interestingly, an increased risk of the time-to-first onset of asthma was found for the AIT group. 

In conclusion, data from long-term RWE studies demonstrate that AIT with grass pollen, tree pollen and mite allergens reduced use of asthma medication and progression of asthma. In patients with allergic rhinitis treated with grass pollen and tree pollen AIT the onset of new asthma is reduced. 

### AIT to treat pediatric asthma 

Despite a smaller database, it was also demonstrated in children that AIT improves not only symptoms of allergic rhinitis. Also in children, SCIT and SLIT reduce asthma symptoms, asthma medication (especially inhalant glucocorticoids), and bronchial hyperreactivity, enhance quality of life, improve lung function, reduce the incidence of asthma exacerbations, and decrease the risk of new onset asthma (reviewed in [23, 24]). Several meta-analyses summarized short-term effects in children like improving asthma control, reducing medication and symptom scores. Conclusive data on long-term efficacy from prospective randomized trials are rare. A small retrospective study evaluated children with asthma 9 years after discontinuation of a SCIT with HDM and grass pollen extract. Children who received an AIT are 3 time less at risk of frequent asthma symptoms compared to those who received no AIT [25]. Indeed, in RWE studies (see above) long-term efficacy could be demonstrated for SCIT and SLIT also for children (see above). 

## Discussion 

RCTs with HDM and pollen extracts in children and adults provide evidence for the efficacy of AIT in patients suffering from asthma. However, most RCTs included subjects with allergic rhinitis with or without allergic asthma, and rhinitis endpoints were the primary outcome parameters. Only a few trials set their focus on allergic asthma. Real-world studies confirm RCT results on broader patient populations and add data of long-term effectiveness and long-term safety. Overall, it has been demonstrated that AIT:

- Reduces asthma symptoms
- Reduces asthma medication and the dose of ics needed to control asthma
- Reduces bronchial hyperactivity
- Reduces the number of asthma exacerbations
- Increases FEV_1 _
- Reduces asthma incidence

SCIT and SLIT are efficacious without principal superiority of one of the administration routes. The allergen peak dose seems to be important for the magnitude of efficacy; therapy duration and the cumulative allergen dose seem to be important for long-term effects [26]. For AIT in the daily routine, allergen extracts with proven efficacy and safety should be preferred, and the treatment should be offered to all suitable allergic rhinitis and asthma patients because of its unique disease-modifying effects. 

## Funding 

None. 

## Conflict of interest 

T. Ankermann reports fees from Abbvie, Allergopharma, Chiesi, Engelhardt, Infectopharm, Novartis, Nutricia, and Sanofi. 

R. Brehler reports fees and/or research support from ALK, Allergopharma, Allmiral, AstraZeneca, Behring, Bencard, GSK, HAL, Leti, Lofarma, MedUpdate, Merck, Novartis, Omnicuris, Sanofi, Stallergenes, TAkeda, Thermo-Fischer. Biotech Tools, Circassia, Genentech. 
